# Precision or adaptability? Contrasting effects of linear and non-linear pedagogy models in handball instruction

**DOI:** 10.3389/fspor.2025.1673424

**Published:** 2025-10-02

**Authors:** Sebastián Espoz-Lazo, Masoud Rahmati Arani, Claudio Hinojosa-Torres, Claudio Farías-Valenzuela, Jalal Dehghani Zadeh

**Affiliations:** 1 Facultad de Educación, Pontificia Universidad Católica de Chile, Santiago, Chile; 2 Faculty of Sport Sciences, Urmia University, Urmia, Iran; 3 Facultad de Educación y Ciencias Sociales, Universidad Andres Bello, Viña del Mar, Chile; 4 Escuela de Ciencias de la Actividad Física, Universidad de Las Américas, Santiago, Chile

**Keywords:** novice, non-linear pedagogy, traditional pedagogy, observational analysis, sport education

## Abstract

**Introduction:**

Research in physical education increasingly compares linear and non-linear teaching for invasion games. The present study examined how these frameworks affect technical execution and game-based decision-making in handball.

**Methods:**

A quasi-experimental pretest–posttest study (5 weeks) assigned students to a Linear model (progressive, repetitive practice) or to Non-Linear Pedagogy (ecological dynamics, task representativeness). Technical skills were measured with the Zinn Handball Skills Battery and the Johanson Ability Test; tactical performance with the Game Performance Assessment Instrument in representative scenarios. Inter-rater reliability was established among independent observers.

**Results:**

Both groups improved technical execution from pre- to post-test. The Linear group showed greater gains in precision-based tasks (passing, shooting), whereas the Non-Linear group improved more in tactical dimensions (decision-making, off-the-ball support). ANOVA indicated significant main effects of time but no group × time interaction; intra- and inter-group contrasts revealed differentiated developmental patterns.

**Discussion:**

Findings suggest complementary strengths: linear instruction favors technical refinement, whereas non-linear approaches promote adaptability, perception–action coupling, and context-sensitive responses.

**Conclusion:**

Educators should adopt flexible, learner-centred programming integrating both models to align with learners' developmental needs.

## 1 Introduction

Handball is a fast-paced, high-intensity team sport characterized by rapid transitions between offensive and defensive phases, constant positional readjustments, and the demand for perceptual and cognitive agility in decision-making. The game unfolds within a highly dynamic environment in which players must adapt continuously to emerging spatial, temporal, and tactical conditions. These adaptations require a high level of communication, coordination, and mutual regulation among teammates, as the success of collective actions depends on the capacity to perceive affordances and act upon them in real time (1). Unlike more predictable or structured sports, such as track and field or swimming, handball presents scenarios where split-second decisions and motor responses can drastically shift the course of play. As such, it is widely recognized as a paradigmatic example of a complex and interactive system in sport (2).

From a technical-tactical perspective, performance in handball emerges through the integration of individual motor competencies and the collective capacity to organize space and time effectively in response to the opponent's behaviour. Offensive sequences are not merely the product of rehearsed patterns but arise from players' ability to explore and exploit the dynamic configurations of defenders and available teammates. Similarly, defensive systems adjust through anticipatory movements and shared interpretations of the attacking team's intentions, requiring a high degree of synchronicity and mutual awareness (3, 4). These coordinated responses are rarely the result of direct instructions during the game; instead, they manifest as emergent properties of the system through processes of self-organization, shaped by prior experience, communicative cues, and tactical adaptation (5). The non-linear nature of these interactions implies that minor modifications, such as a subtle change in a player's position, can produce disproportionately large effects on the system's behaviour, a phenomenon previously discussed in the context of attractors and phase transitions in team sports (2).

However, in contrast to ecological and dynamic systems-based approaches, some traditional perspectives in sport pedagogy have conceptualized handball as a structured set of discrete motor actions. These models emphasize technical repetition, sequential progressions, and the mechanistic assembly of isolated skills into predictable patterns of play (6, 7). From this viewpoint, handball performance is optimized through cyclical training routines that aim to automate technical execution, often neglecting the emergent and adaptive nature of tactical behaviour in game contexts (8, 9). Such frameworks align with linear and reductionist paradigms, where the sport is viewed as a system of interlocking parts operating in fixed, repetitive cycles (10, 11).

Despite the inherently adaptive and fluid nature of handball, traditional training methodologies have predominantly relied on mechanistic and linear pedagogical models (12, 13). These approaches conceive learning as a process of internalizing ideal motor patterns through prescriptive instruction and repetition of isolated skills, often detached from the perceptual and tactical demands of actual gameplay (14, 15). Training sessions are typically organized in a sequential manner, progressing from simple, technique-focused drills to more complex, yet still constrained, tasks aimed at refining biomechanical precision (1). This logic assumes that correct execution in closed environments will naturally transfer to open, unpredictable game contexts. However, such an assumption neglects the non-linearity, variability, and emergent nature of motor behavior in team sports, raising concerns about the ecological validity and transferability of skills developed through decontextualized training regimes (3, 4, 13).

In response to these limitations, non-linear pedagogical approaches have gained prominence as alternative frameworks for designing ecologically valid learning environments. Grounded in ecological dynamics, complex systems theory, and dynamical systems modelling, non-linear pedagogy (NLP) proposes that learning emerges through the manipulation of constraints that shape players' exploratory behaviour (16, 17). Rather than isolating technical elements, NLP integrates perceptual, cognitive, and motor demands into representative learning tasks, encouraging learners to discover functional movement solutions through guided variability and problem-solving. This approach aligns with the view that motor learning is a process of attunement to relevant environmental information, where decision-making co-evolves with action through situated interaction (18, 19). By emphasizing autonomy, adaptability, and creativity, NLP seeks not only to enhance performance but also to foster deeper engagement and more meaningful learning experiences. A compelling example of NLP application in handball is provided by Práxedes et al. (20), who implemented a teaching program based on the Constraint-Led Approach with under-12 male players. Through task constraints and modified game situations, players were encouraged to adapt their actions and make tactical decisions in representative contexts. The results showed significant improvements in decision-making and tactical execution, as players learned to identify affordances and coordinate behaviour under game-relevant pressures. This study reinforces the potential of NLP to develop tactical understanding and functional performance through experiential and situated learning processes.

Recent empirical evidence also reinforces the practical value of non-linear pedagogy in handball. For instance, Práxedes et al. (20) demonstrated that constraint-led teaching programs with youth players significantly enhanced tactical decision-making and adaptability, highlighting the potential of representative learning tasks to foster functional behaviour. Similarly, Chow et al. (14) and Renshaw et al. (21) argued that constraint-based environments facilitate perception–action coupling and adaptive learning processes that cannot be achieved through repetitive, decontextualized drills. These contributions support the theoretical rationale for comparing linear and non-linear pedagogical models in complex invasion games such as handball.

Furthermore, research in team sports has shown that ecological and non-linear approaches not only benefit individual skill learning but also promote coordination of collective tactical actions. Correia et al. (22), for example, reported that representative learning designs improve the transferability of tactical behaviours across contexts. This evidence suggests that non-linear frameworks may provide a more comprehensive understanding of skill acquisition in handball, particularly in educational and developmental settings.

Nevertheless, empirical support for non-linear pedagogy is not uniformly conclusive. For instance, Bagheri et al. (23) examined the effects of a non-linear intervention on the motor creativity of sixth-grade girls in a school setting. Contrary to expectations, the study found no statistically significant improvements in creative motor behaviour, raising questions about the conditions necessary for NLP effectiveness. The authors attributed this to reduced physical activity due to pandemic restrictions, irregular attendance, and limited instructional continuity, factors that may have undermined the intervention's impact. These findings underscore that the success of non-linear models depends not only on their theoretical design but also on the contextual and organizational variables shaping the learning environment. Despite growing interest in NLP, research exploring its application in empirical contexts, particularly among university students, remains limited. While the majority of the NLP studies with practical implications have focused on youth and school populations, the transition of ecological approaches into higher education has yet to be adequately investigated (3, 4). This is particularly relevant in teacher education programs, where pedagogical models not only impact students' own learning but also influence their future teaching practices. The lack of comparative studies in this domain constrains our understanding of how to optimize initial handball learning experiences for adult novices.

Given the complexity of handball and the theoretical contrast between Linear and Non-Linear pedagogies, it is essential to examine how each model influences technical and tactical learning. Traditional approaches may provide structure and clarity for beginners but risk oversimplifying the dynamic and interactive nature of gameplay. Modern approaches, in contrast, promote exploration and adaptation but may require greater instructional sensitivity and support to be effective. A comparative analysis that considers both performance outcomes and tactical understanding is thus needed to inform evidence-based practices in sport instruction and physical education. Such research contributes not only to pedagogical theory but also to the practical challenge of designing inclusive, engaging, and developmentally appropriate learning environments for novice players. Therefore, the aim of this study is to compare the effects of a linear methodology and a non-linear model on technical and tactical outcomes in university students with no prior experience in handball.

## 2 Material & method

### 2.1 Participants

A total of 23 undergraduate students (Age 21 ± 1.2) enrolled in an initial physical education teacher training program at the Urmia University participated in the study. Participants were recruited using a non-probability convenience sampling approach. None of them had previous experience in handball or had received formal instruction in the sport prior to the intervention. All participants voluntarily agreed to take part in the study and provided informed consent. The study was approved by the institutional ethics committee, and all procedures complied with the principles of the Declaration of Helsinki (24).

### 2.2 Design and procedure

A quasi-experimental pretest–posttest design with two non-randomized groups was employed. Participants were recruited using a non-probability convenience sampling approach. Performance variables were evaluated using both technical tests (ZHSB and Johanson Ability Test) and tactical assessment (GPAI).

Participants were assigned to groups based on course enrollment: One group taught using a Linear Pedagogy approach (LP), while the other received instruction through a Non-Linear Pedagogy based on the Constraint-Led Approach (CLA). Both groups underwent 10 training sessions over a five-weeks period (two sessions per week), with each session lasting approximately 90 min. The sessions focused on basic handball skills and tactical understanding, but differed in instructional methodology according to the assigned pedagogical model (Table 1).

**Table 1:** Summarized planification for linear pedagogy (LP) & constrain led approach (CLA) training sessions.

Type of training	Session	Topic	Objective	Main activities
LP	1	Dribbling	Basic dribbling	Isolated drills with linear progression
	2		Ball control	Linear and zigzag dribbling drills
	3	Passing	Basic passing	Unopposed partner passing
	4		Passing accuracy	Targeted triangle passing
	5	Shooting	Basic shooting	Unopposed goal shooting
	6		Shooting on the move	Dynamic shooting from 6 m
	7	Receiving	Basic reception	Stationary and dynamic reception
	8		Reception on the move	Running reception with pass
	9	Simple Situations	Isolated fundamentals	Technical skill circuits
	10	Small-Sided Games	Fundamentals in game play	3v3 with technical fundamentals
CLA	1	Dribbling	Explore dribbling	Dribbling-focused conditioned game
	2		Adapt dribbling	Adaptive space/rule modification
	3	Passing	Explore passing	Passing variety games
	4		Adapt passing	Passing under variable contexts
	5	Shooting	Explore shooting	Targeted shooting game
	6		Adapt shooting	Shooting under pressure
	7	Reception	Explore reception	Reception variability games
	8		Adapt reception	Speed/space adaptation tasks
	9	Complex situations	Solve complex play	Small-sided with multiple targets
	10	Small-sided game	Tactical adaptation	Adaptive small-sided game

Regarding the intervention, the LP group followed a traditional training approach that emphasized technical drills, isolated skill repetition, and progressively increasing task complexity. In contrast, the CLA group engaged in modified games and representative learning tasks designed to manipulate task constraints and foster perceptual-motor exploration and tactical adaptation.

Pre- and post-intervention evaluations were conducted to assess changes in technical performance and tactical behaviour.

Data collection was carried out under standardized conditions for both groups. Technical performance tests (ZHSB and Johanson Ability Test) were administered individually in controlled practice settings, with participants performing the required skills on a regulation handball court (40 × 20 m) using official handballs. Tactical performance (GPAI) was assessed in representative small-sided game situations (3 vs. 3 with goalkeepers) designed to replicate formal game constraints while maintaining experimental control. Each tactical scenario lasted two 8 min periods and was video recorded for subsequent coding by two independent observers.

To ensure reliability and facilitate replication, all participants completed the same sequence of tasks, and the two observers were previously trained in the use of the GPAI coding system. These procedures were designed to strengthen ecological validity while maintaining replicability across both pedagogical conditions.

Although no pilot study was conducted, the training load, intensity, duration, and volume were carefully planned according to the professional expertise of the research team, who are experienced handball coaches and former professional players. This practical background ensured that the intervention was both appropriate for novice learners and aligned with the demands of handball training in educational settings.

### 2.3 Instruments

#### 2.3.1 Technical performance

Both groups were assessed with two complementary approaches. Technical performance was evaluated using the Zinn Handball Skills Battery (passing and shooting) and the Johanson Ability Test (25, 26). In addition, tactical performance was evaluated for both groups using the Game Performance Assessment Instrument (GPAI), focusing on skill execution, support, and decision-making in representative game situations (27). This combined approach ensured that both technical and tactical dimensions were captured across groups.

#### 2.3.2 Data analysis

All statistical analyses were performed using IBM SPSS Statistics® version 28.0.0.0 (190). The analytical approach was designed to align with the nature of the data and the objectives of the study.

For the linear performance tests, descriptive statistics were computed to summarize central tendency and dispersion across pre- and post-intervention stages. To explore relationships among continuous motor variables (e.g., execution time and shot accuracy), Pearson's correlation coefficient was used, given its robustness in detecting linear associations among interval-level variables when normality assumptions are reasonably met (28).

To examine the effects of the training interventions, 2 × 2 mixed-design ANOVAs were conducted with time (pre vs. post) as a within-subject factor and group (CLA vs. LP) as a between-subject factor. This model allows for testing both main effects and interaction effects, providing insight into within-group changes over time and between-group differences in performance trajectories (29). Partial eta squared (*η*²_p_) was used to estimate effect sizes, and statistical significance was set at *p* < .05.

Given that the observational variables derived from video coding were ordinal and based on subjective ratings, non-parametric methods were employed. Inter-rater reliability was assessed using Spearman's rank-order correlation coefficient, a suitable statistic for evaluating monotonic agreement between coders when data are not normally distributed (30, 31).

To evaluate intra-group improvements from pre- to post-intervention within each training condition, the Wilcoxon signed-rank test was applied. This non-parametric alternative to the paired *t*-test is recommended for repeated measures designs when data are ordinal or deviate from normality (32).

Finally, post-intervention comparisons between groups were analysed using the Mann–Whitney *U*-test. This test serves as a robust alternative to the independent samples *t*-test when assumptions of normality or homogeneity of variance are violated, particularly for small samples or ordinal data (33). All statistical tests were two-tailed, and alpha was set at 0.05.

## 3 Results

### 3.1 Descriptive analysis of linear tests (pre-intervention)

At the pre-intervention stage, both the CLA and LP groups displayed comparable profiles across the linear test variables. Although slight differences emerged, for instance, in passing or shooting performance, these were not large enough to suggest meaningful disparities before the intervention. This homogeneity in baseline performance supports the validity of the group comparison in the subsequent stages (Table 2).

**Table 2:** Results of Zinn handball skills battery & Johanson ability test.

Variables	CLA					LP				
	N	Min	Max	Mean	SD	N	Min	Max	Mean	SD
Passing Time	12	11.33	13.74	12.40	0.720	11	13.02	15.36	14.19	0.77
Passing Score	12	26	43	38.25	4.65	11	34	47	42.27	4.05
Standing Shot	12	0	12	7.25	3.30	11	4	16	8.27	3.49
Jumping Shot	12	0	14	8.67	4.05	11	6	18	11.64	4.31
Ability test	12	20	21	20.42	0.51	11	20	21	20.36	0.50

Pearson correlation analyses revealed consistent relationships among variables. A strong and statistically significant negative correlation was observed between execution time and passing score (*r* = −.503, *p* = .014), suggesting that participants who performed the task more quickly tended to be more accurate. Additionally, a strong positive correlation was found between standing and jumping shot scores (*r* = .733, *p* < .001), indicating a shared underlying proficiency in shooting ability.

### 3.2 Descriptive analysis of linear tests (post-intervention)

Post-intervention data showed improvements across all linear test variables for both groups. Participants in the CLA group improved in execution speed and maintained solid technical performance, whereas those in the LP group demonstrated increases particularly in accuracy-based metrics. The overall gains reflect a positive response to both instructional approaches (Table 3).

**Table 3:** Results of Zinn handball skills battery & Johanson ability test post intervention.

Variables	CLA					LP				
	N	Min	Max	Mean	SD	N	Min	Max	Mean	SD
Passing Time	12	9.71	12.13	11.20	0.71	11	10.00	15.20	13.33	1.63
Passing Score	12	30	48	41.33	4.79	11	40	49	45.91	2.42
Standing Shot	12	8	14	11.25	2.17	11	7	16	10.55	2.46
Jumping Shot	12	10	18	14.17	2.25	11	10	20	15.09	3.41
Abillity test	12	25	33	29.17	2.48	11	25	31	28.09	2.34

Correlations between variables remained consistent. The inverse relationship between passing time and accuracy was still evident (*r* = −.511, *p* = .012), as was the positive association between standing and jumping shot scores (*r* = .697, *p* < .001). These results reinforce the internal consistency of performance profiles post-intervention.

### 3.3 Inferential analysis of linear tests

To examine the effects of the training interventions, a 2 × 2 mixed-design ANOVA was conducted for each performance variable. Results revealed significant main effects of time across all tasks, indicating that both groups improved their performance from pre- to post-intervention. Group effects were also found in several variables, with the LP group showing greater gains in tasks traditionally associated with repetition and mechanical precision, such as passing and shots (Table 4). However, no significant interaction effects were found, suggesting that both instructional approaches produced similar developmental trends over time.

**Table 4:** Results of ANOVA on Zinn handball skills battery.

Variable	Effect	*F*(1, 21)	*P*-value	*η* ^2^_p_
Execution time	Time	39.27	<.001	.651
	Group	17.58	.001	.456
	Time × Group	2.40	.137	.103
Passing score	Time	70.31	<.001	.770
	Group	6.23	.021	.229
	Time × Group	0.33	.571	.016
Standing shot	Time	15.93	.001	.431
	Group	15.82	.001	.430
	Time × Group	0.34	.566	.016
Jumping shot	Time	20.92	<.001	.499
	Group	0.48	.496	.022
	Time × Group	2.03	.169	.088

Same situation was found in the dribbling task. Table 5 shows significant main effects of both time and group. The LP group performed better overall, and both groups improved from pre to post. However, no significant interaction was detected, indicating similar improvement trajectories regardless of instructional condition.

**Table 5:** Results of ANOVA on Johanson ability test.

Dependent Variable	Effect	F	*P*	*η*²_p_
Abillity test	Time	31.23	<.001	.598
	Group	13.27	.002	.387
	Time × Group	0.47	.500	.022

These results support the idea that both interventions were effective in enhancing technical aspects of performance. While the Linear approach seemed to provide advantages in tasks favouring execution consistency, the Non-Linear approach also led to substantial improvements, particularly in speed and adaptability.

### 3.4 Observer agreement in game performance assessment instrument (GPAI)

Spearman's rank correlation coefficients between the two independent observers demonstrated moderate to strong and statistically significant relationships across all assessed dimensions (technical skill execution, decision-making, and support), at both the pre- and post-intervention stages (Table 6). These consistent correlations confirm the reliability of the observational coding system and suggest a robust level of agreement between raters, supporting the validity of the data collected.

**Table 6:** Coefficients results between the two observers.

Variable	Spearman's	*P*
Skill Pre	0.419	0.004
Skill_post (Ob1 vs. Ob2)	0.519	<0.001
Decision_pre (Ob1 vs. Ob2)	0.419	0.004
Decision_post (Ob1 vs. Ob2)	0.649	<0.001
Support_pre (Ob1 vs. Ob2)	0.295	0.046
Support_post (Ob1 vs. Ob2)	0.581	<0.001

### 3.5 Intragroup analysis: nonlinear performance

Wilcoxon signed-rank tests were used to evaluate within-group changes in tactical behaviour as assessed through nonlinear tests. Results indicated statistically significant improvements in all dimensions for the CLA group following the intervention. Participants exhibited clear progress in skill execution, decision-making, and tactical support, with the majority showing positive rank changes. The LP group also showed significant, albeit more modest, improvements in several areas. However, tactical support in particular revealed a smaller effect size and a higher frequency of tied ranks, especially in Tactical Situation 1 (Table 7). These results suggest that while both interventions yielded positive outcomes, the nonlinear pedagogy produced broader and more consistent effects across tactical dimensions.

**Table 7:** Results of Wilcoxon signed-rank tests.

Group	Ability	TS 1			TS 2		
		N	Z	*P* value	N	Z	*P* value
CLA	Skill post vs. Skill pre	24	−4.4	<.001	24	−4.167	<.001
	Decision post vs. Decision pre	24	−4.242	<.001	24	−4.291	<.001
	Support post vs. Support pre	24	−3.578	<.001	24	−3.947	<.001
LP	Skill post vs. Skill pre	22	−3.5	<.001	22	−0.207	0.001
	Decision post vs. Decision Pre	22	−2.982	0.003	22	−3.606	<.001
	Support post vs. Support pre	22	−2.309	0.021	22	−3.207	0.001

TS, tactical situation.

### 3.6 Intergroup analysis: nonlinear performance

Mann–Whitney *U*-tests were conducted to compare post-intervention performance between the CLA and LP groups in both tactical situations. The results showed statistically significant differences in favour of the CLA group across all three dimensions (skill execution, decision-making, and support) (Table 8). These differences were consistent in both tactical scenarios, reinforcing the greater impact of the nonlinear pedagogical model in enhancing adaptive and context-sensitive aspects of performance.

**Table 8:** Results for Mann–Whitney (intergroup analysis).

Context		Group	N	Mann–Witney	*P*
TS 1	Skill	CLA	24	108	<.001
		LP	22		
	Decision	CLA	24	96	<.001
		LP	22		
	Support	CLA	24	154.5	0.007
		LP	22		
TS 2	Skill	CLA	24	93.5	<.001
		LP	22		
	Decision	CLA	24	99.5	<.001
		LP	22		
	Support	CLA	24	148.5	0.004
		LP	22		

TS, tactical situation.

## 4 Discussion

The primary objective of this study was to compare the effects of two pedagogical models—Linear Pedagogy (LP) and Non-Linear Pedagogy (NLP), specifically the Constraint-Led Approach (CLA)—on the development of technical and tactical skills in university students with no prior handball experience. By integrating linear performance tests and game-based observational tools, the study aimed to assess not only technical execution in isolated contexts but also players' decision-making and tactical support in representative, game-like scenarios. This dual-layered approach, supported by a quasi-experimental design, seeks to contribute to a deeper understanding of how pedagogical structures shape learning outcomes in novice adult learners, particularly in initial teacher education programs.

Baseline data revealed comparable performance levels across all measured technical variables in both groups, lending internal validity to the group comparisons. Minor differences observed—such as slightly higher passing and shooting scores in the LP group—were not sufficient to suggest any meaningful pre-existing advantage. This general parity is especially relevant in quasi-experimental designs where random allocation is absent, as it reduces the risk of confounding variables influencing the outcomes and thus enhances the interpretability of the post-intervention effects (34).

The correlational analysis at pre-test added valuable insight into the interrelation of technical variables. The significant inverse relationship between execution time and passing accuracy suggests a coordinated integration of speed and precision in ball-handling, consistent with prior research in similar tasks (35). Similarly, the strong positive correlation between standing and jumping shot scores implies shared underlying biomechanical or perceptual-motor proficiencies. These patterns validate the multidimensional structure of the test battery and reinforce the importance of composite skill assessments in the evaluation of motor competence. By the other hand, the post-intervention analyses indicated substantial improvements across all technical tests for both groups. The CLA group showed notable enhancements in execution speed while maintaining technical accuracy, supporting the view that representative learning environments foster adaptive movement solutions (21). Meanwhile, the LP group demonstrated increased accuracy in passing and shooting, aligning with expectations from structured, repetition-based training models (36). These outcomes suggest that although both methods are effective, they may prioritize different aspects of skill development.

The persistence of pre- to post-test correlations further supports the internal structure of motor performance. The continued inverse correlation between passing time and accuracy reflects an enduring balance between control and speed, a critical feature in handball performance (35). Additionally, the ongoing positive relationship between shot modalities suggests a degree of skill transfer between actions, reinforcing the notion of generalizable movement principles across related tasks (43).

Inferential analyses confirmed significant main effects of time across all assessed variables, indicating that both pedagogical models facilitated technical improvements. This is consistent with research showing that structured physical education interventions, regardless of pedagogical orientation, can yield measurable benefits in early stages of skill acquisition (35, 37). The absence of interaction effects suggests comparable developmental trends across groups, though with nuanced differences in the nature of the gains. The presence of significant group effects in accuracy-based tasks (e.g., passing and standing shots) points to the LP group's advantage in exercises emphasizing repetition and biomechanical stability. These results resonate with critiques of linear instruction that, while limited in fostering adaptability, often excel in refining isolated technical performance in controlled environments (21, 36). Conversely, the CLA group's lack of superiority in such variables may reflect its orientation toward exploration rather than repetition.

Notably, the CLA group showed superior improvements in execution time, which may reflect increased fluency and perceptual-motor integration. These outcomes align with the theoretical underpinnings of non-linear pedagogy, which views skill learning as an emergent process shaped by the interaction of task, environment, and individual constraints (14). In dynamic sports like handball, where rigid performance can be a liability, this adaptability may offer long-term advantages.

Observer agreement in the GPAI analysis was consistently high, confirming the reliability of the observational protocol. Spearman correlations revealed statistically significant inter-observer consistency across all variables and time points. This is consistent with findings that emphasize the value of well-trained observers and robust coding protocols in behavioral analysis (38–40). High observer agreement not only supports the integrity of the dataset but also strengthens the validity of the conclusions drawn from it.

Within-group analyses revealed substantial improvements for the CLA group across all tactical dimensions. Skill execution, decision-making, and support behaviours improved significantly, highlighting the effectiveness of constraint-led environments in facilitating emergent, context-sensitive learning. These results echo previous findings on the benefits of representative task design for promoting perception-action coupling and strategic autonomy (20, 21). At the same time, the LP group also showed within-group improvements, though these were less pronounced. In particular, tactical support—especially in the first game situation—showed lower effect sizes and more tied ranks. This may reflect the limitations of linear instructional formats in fostering the coordinated, context-responsive behaviours required for tactical synchronization, which are often shaped through interaction rather than instruction (14, 41).

Between-group comparisons following the intervention confirmed the CLA group's superiority in tactical performance. Across both game situations, participants in the nonlinear group outperformed their LP counterparts in decision-making, support, and technical execution. This supports a growing body of literature advocating for nonlinear pedagogical approaches as more effective in fostering functional, adaptive behavior in sport (14, 21).

The robustness of these differences across both tactical scenarios further emphasizes the CLA's value in supporting coherent, transferable behaviors across contexts. Prior research in team sports has shown that constraint-based learning not only enhances individual decision-making but also improves coordination of collective tactical actions (22, 42). In contrast, the LP group's technical improvements did not appear to translate effectively to dynamic gameplay contexts.

This study is not without limitations. The small sample size (*N* = 23) and the use of a convenience, non-randomized allocation limit the generalizability of the findings and prevent strong causal inferences. In addition, the relatively short duration of the intervention (five weeks, ten sessions) may not fully capture long-term learning effects. Although validated instruments were employed (ZHSB, Johanson Ability Test, GPAI), the alignment between training tasks and evaluation tools was not complete, which may have constrained the sensitivity of the assessment. Regarding the statistical analyses, while non-parametric tests were appropriate for the distribution of the data, no formal power analysis was conducted and effect sizes were not reported, which reduces the robustness of the inferences. Future research with larger randomized samples, longer interventions, closer alignment between practice tasks and evaluation, and the inclusion of power and effect size analyses is recommended to strengthen the evidence base.

Taken together, the findings of this study highlight the multidimensional nature of learning in sport, particularly in complex environments like handball. The improvements observed across both pedagogical models suggest that each can contribute to the development of technical and tactical competencies, albeit through different mechanisms. The CLA appears to facilitate a more integrated, context-driven learning process, while the LP offers structure and repetition that may support technical stabilization. The complementarity of these approaches invites further inquiry into how pedagogical design can be tailored to the learner's developmental stage and the performance context. Ultimately, rather than positioning one pedagogical model as inherently superior to the other, these results advocate for a more flexible and responsive instructional approach. Teachers and coaches may benefit from navigating between linear and non-linear strategies depending on learners' prior experience, the complexity of the task, and the phase of development or competition. A blended model that recognizes when to prioritize structure and when to encourage exploration may offer the most promising route for fostering both technical mastery and tactical intelligence in team sport settings.

In conclusion, this study demonstrates that both Linear Pedagogy (LP) and the Constraint-Led Approach (CLA) contribute to the development of novice handball players, although through different mechanisms. LP supports technical refinement and consistency, while CLA fosters adaptability, tactical awareness, and decision-making under game-like conditions. For teachers and coaches, these findings highlight the practical value of integrating both approaches strategically: linear drills may be useful to introduce and stabilize basic techniques, whereas non-linear, game-representative tasks can accelerate tactical understanding and collective coordination. By adopting a flexible and learner-centered methodology, practitioners can better respond to the developmental needs of their students and athletes, ultimately enhancing the quality of handball instruction in both educational and training contexts.

## Data Availability

The raw data supporting the conclusions of this article will be made available by the authors, without undue reservation.
